# Glucosinolate extract from radish (*Raphanus sativus* L.) seed attenuates high-fat diet-induced obesity: insights into gut microbiota and fecal metabolites

**DOI:** 10.3389/fnut.2024.1442535

**Published:** 2024-08-08

**Authors:** Quanfeng Zhu, Peng Zhang, Daqun Liu, Leilei Tang, Jiawen Yu, Chengcheng Zhang, Guojun Jiang

**Affiliations:** ^1^Affiliated Xiaoshan Hospital, Hangzhou Normal University, Hangzhou, China; ^2^Food Science Institute, Zhejiang Academy of Agricultural Sciences, Hangzhou, China

**Keywords:** radish seeds, glucosinolate, obesity, gut microbiota, fecal metabolome

## Abstract

**Background:**

Radish seed is a functional food with many beneficial health effects. Glucosinolates are characteristic components in radish seed that can be transformed into bioactive isothiocyanates by gut microbiota.

**Objective:**

The present study aims to assess anti-obesity efficacy of radish seed glucosinolates (RSGs) and explored the underlying mechanisms with a focus on gut microbiota and fecal metabolome.

**Methods:**

High-fat diet-induced obese mice were supplemented with different doses of RSGs extract for 8 weeks. Changes in body weight, serum lipid, alanine aminotransferase (ALT), and aspartate aminotransferase (AST) levels; and pathological changes in the liver and adipose tissue were examined. Fecal metabolome and 16S rRNA gene sequencing were used to analyze alterations in fecal metabolite abundance and the gut microbiota, respectively.

**Results and conclusion:**

Results showed that RSG extract prevented weight gain and decreased serum lipid, ALT, AST levels and lipid deposition in liver and epididymal adipocytes in obese mice. Treatment with RSG extract also increased gut microbiota diversity and altered the dominant bacteria genera in the gut microbiota, decreasing the abundance of *Faecalibaculum* and increasing the abundance of *Allobaculum*, *Romboutsia*, *Turicibacter*, and *Akkermansia*. Fecal metabolome results identified 570 differentially abundant metabolites, of which glucosinolate degradation products, such as sulforaphene and 7-methylsulfinylheptyl isothiocyanate, were significantly upregulated after RSG extract intervention. Furthermore, enrichment analysis of metabolic pathways showed that the anti-obesity effects of RSG extract may be mediated by alterations in bile secretion, fat digestion and absorption, and biosynthesis of plant secondary metabolites. Overall, RSG extract can inhibit the development of obesity, and the obesity-alleviating effects of RSG are related to alternative regulation of the gut microbiota and glucosinolate metabolites.

## 1 Introduction

Over the past several decades, obesity, a chronic metabolic disease, has become a public health problem around the world. Obesity can be triggered by a combination of genetics, lifestyle, and other environmental factors and is characterized by abnormal energy metabolism and excessive fat deposition in the body (1). Due to modern diets (e.g., high fat, high sugar, ultraprocessed foods, etc.) and more sedentary lifestyles, the prevalence of obesity and its resulting complications, such as hyperlipidemia, hypertension, cardiovascular disease, and fatty liver, is increasing each year (2, 3). To prevent subsequent health issues caused by obesity and resulting metabolic diseases, it is important to develop safe and effective strategies to help prevent and alleviate excessive weight gain.

Plant-derived functional foods are remarkably effective as weight loss supplements, fat-reducing agents, and treatments for chronic metabolic diseases, and, importantly, these products produce fewer toxic side effects than chemical medicine (4–6). Radish (*Raphanus sativus* L.) seed (called “Lai Fu-zi” in Chinese) is widely used in Chinese herbal medicine and functional foods. Radish seeds are rich in glucosinolates, alkaloids, terpenoids, polyphenols, and previous studies have suggested that radish seed extract exhibits notable efficacy in anti-tumor (7), anti-inflammatory (8), anti-diabetes (9), and anti-obesity (10). Particularly, the glucosinolate components can regulate lipid metabolism and ameliorate metabolic syndrome (11, 12). For example, glucoraphenin, the main glucosinolates component in radish, can reduce weight gain and hepatic lipid accumulation and improve the levels of serum lipid levels in high-fat diet-induced obesity mice (13). Glucoraphanin, which is abundant in broccoli seeds, also has good potential for obesity prevention via regulating genes involved in lipid metabolism (FAS, PPARα, CPT1, and ACOX) in obese mice (14). These studies suggested the potential of glucosinolates in promoting weight loss and lowering lipid levels.

The gut microbiota is a critical mediator of obesity, as the composition of the gut microbiota affects host lipid metabolism (15, 16). Development of obesity is typically accompanied by a marked imbalance in the abundance of specific bacteria in the gut microbiota and a decrease in gut microbiota diversity, for example, shifts in the abundance of Firmicutes and Bacteroides, the two main phyla of the gut microbiota (17, 18). Notably, many natural active ingredients that are used to treat obesity regulate the gut microbiota, and the composition of the gut microbiota could impact the bioavailability of active substances (19, 20). After oral intake, the active ingredients can be transformed into functional metabolites via the gut microbiota and absorbed from the intestine into the blood (21). For example, the gut microbiota can degrade glucosinolates, breaking them down into active isothiocyanate-like products, which in turn exert biological activity (22).

To date, the obesity-alleviating effects of radish seed glucosinolates (RSGs) remain unknown, and the interaction between gut microbiota and metabolites in high-fat diet-induced obese mice supplemented with RSGs still unclear. To fill this knowledge gap, we established a mouse model of obesity by feeding C56BL/6J mice a high-fat diet (HFD), administering RSG extract, and observing changes in body weight and serum lipid levels. Furthermore, 16S rRNA gene sequencing and fecal metabolome were performed to explore the underlying anti-obesity mechanisms of RSGs from the perspective of gut microbiota and fecal metabolites. The results of this study will give a scientific basis for expanding the application of radish seeds in functional foods and nutraceuticals.

## 2 Materials and methods

### 2.1 Preparation of RSG extract

Dried mature seeds of radish were obtained from Beijing TongRenTang Co. Ltd., (Beijing, China). The radish seeds were then ground into a powder and extracted using 60% ethanol with stirring for 30 min in a 70°C water bath. Extracts were then centrifuged at 4°C and 3,000 rpm for 10 min. Polyvinylpolypyrrolidone (PVPP) was added to the supernatant extract at a PVPP:RSG extract ratio of 0.85:100 (wt:vol), stirred for 30 min, and centrifuged for 10 min (4°C, 3,000 rpm) (23). Samples were evaporated using a rotary evaporator, freeze-dried, and stored at −20°C.

### 2.2 Analysis of glucosinolate content in RSG extract

The glucosinolate content in RSG extract was analyzed using methods similar to those described previously (24) and a SCIEX 5500 ultra-high-performance liquid chromatography-triple quadrupole mass spectrometer (AB SCIEX, Massachusetts, United States) coupled with a CSH C18 column (2.1 × 100 mm, 1.7 μm; Waters, Milford MA, United States). Mobile phase A comprised 0.1% (vol/vol) formic acid in 5% (vol/vol) acetonitrile, whereas mobile phase B comprised 0.1% (vol/vol) formic acid in 100% (vol/vol) acetonitrile. The following gradient of elution was used (flow rate = 0.4 mL/min): 0–2.5 min, 0–50% B; 2.5–3.0 min, 50–0% B; 3.0–3.5 min, 0% B. The following scanning mode was used: negative ions, and scanning in multireaction monitoring (MRM) mode. Glucosinolate detection was performed in MRM mode of selected ions at the first and third quadrupole (Table 1). A standard curve was generated with glucoraphanin as the standard to calculate the glucosinolate content.

**Table 1:** Glucosinolates composition in RSG extract and their mass spectrum parameters.

General structure	Glucosinolates	R-groups	CAS	Formula	Q1	Q3	CE (V)
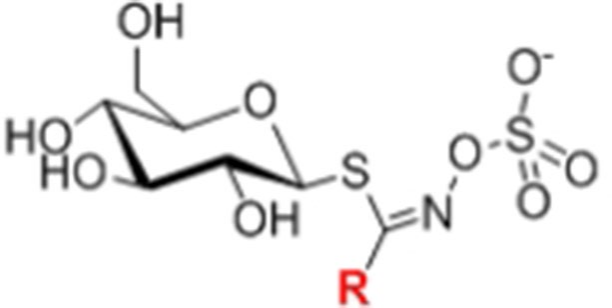	Glucoraphenin	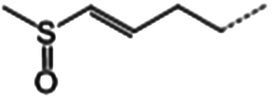	28,463–24-3	C_12_H_21_NO_10_S_3_	434	97	20
	Glucoraphasatin	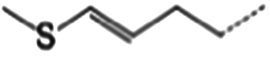	28,463–23-2	C_12_H_21_NO_9_S_3_	418	97	20
	Glucoiberin	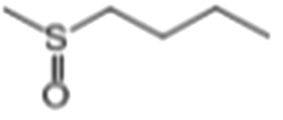	554–88-1	C_11_H_21_NO_10_S_3_	422	97	20
	Glucoiberverin	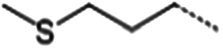	26,888–03-9	C_11_H_20_NO_9_S_3_	406	97	20
	Progoitrin	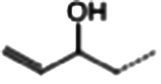	585–95-5	C_11_H_19_NO_10_S_2_	388	97	20
	Glucoraphanin	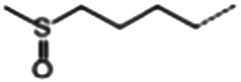	21,414–41-5	C_12_H_23_NO_10_S_3_	436	97	20
	Glucoerucin	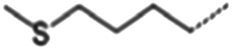	21,973–56-8	C_12_H_23_NO_9_S_3_	420	97	20
	Gluconasturtiin	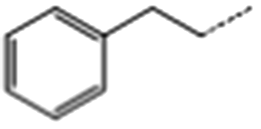	499–30-9	C_15_H_21_NO_9_S_2_	422	97	20
	Glucobrassicin	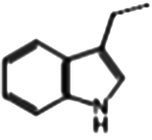	4,356-52-9	C_16_H_20_N_2_O_9_S_2_	446	97	20
	Neoglucobrassicin	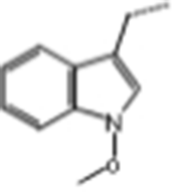	5,187-84-8	C_17_H_22_N_2_O_10_S_2_	477	97	20
	Hydroxyglucobrassicin	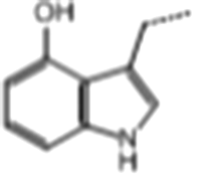	83,327–20-2	C_16_H_20_N_2_O_10_S_2_	463	97	20

### 2.3 Animal experimental design

Seven-week-old male C57BL/6J mice (20 ± 2 g; Shanghai SLAC Laboratory Animal Center, Co., Ltd.) were housed in the Laboratory Animal Center of Zhejiang University of Technology (ZJUT) under specific pathogen-free conditions. Six mice were housed per cage in a 12-h light/12-h dark cycle (8:00–20:00 illumination) at 23 ± 3°C with a humidity of between 45 and 65%. All experiments were performed under strict adherence to internationally accepted principles for the use and care of laboratory animals, and all protocols were approved by the Animal Ethics Committee of ZJUT (ZH20230904008).

High-fat diet-induced obese mice model was established according to a previous study (7). In brief, mice were acclimated for 1 week and divided into five groups (*n* = 6 mice per group): nonobese control mice (CON; fed a low-fat diet containing 10% fat), control-treated obese mice (MOD; fed an HFD containing 45% fat), and RSG-low (RSG-L), RSG-medium (RSG-M), and RSG-high [RSG-H; fed an HFD containing 45% fat and administered daily intragastric doses of 100, 200, and 400 mg per kg (body weight) RSG extract, respectively]. Mice in the CON and MOD groups received equal volumes of normal saline (10 mL/kg, once daily). The nutritional compositions of experimental diets are shown in Table 2. The intervention experiment lasted 8 weeks, and body weight was recorded weekly. The food was changed and weighed daily at regular intervals to calculate food intake.

**Table 2:** Nutritional composition of the experimental diets.

Ingredient	Low-fat diet (g%)	High-fat diet (g%)
Corn starch	29.86	8.48
Maltodextrin	3.32	11.65
Sucrose	33.17	20.14
Soybean oil	2.37	2.91
Lard	1.9	20.68
Cellulose	4.74	5.83
Casein	18.96	23.31
L-cysteine	0.28	0.35
Minerals	4.24	5.23
Vitamins	0.95	1.16
Choline bitartrate	0.19	0.23

After the eighth week, mouse feces were collected and stored at −80°C. One day before the experimental endpoint, the mice were fasted overnight, and cervical dislocation was performed after blood was taken from the eye socket. Perirenal and epididymal fat was collected and weighed, and liver and adipose tissue were dissected and preserved in formalin solution. Serum was isolated by incubating blood samples at 4°C for 2 h, centrifuging for 15 min at 3,000 rpm, and freezing at −80°C.

### 2.4 Serum biochemistry

To assess the impacts of RSG on serum lipid levels, serum samples were probed for triglycerides (TG) and total cholesterol (TC) as well as the individual cholesterols high-density lipoprotein (HDL-C) and low-density lipoprotein (LDL-C). The liver enzymes alanine aminotransferase (ALT) and aspartate aminotransferase (AST) were also analyzed to determine the impacts of RSG on liver health. Each assessment was performed using commercial kits (Nanjing Jiancheng Bioengineering Institute, Nanjing, China), as per the manufacturer’s instructions.

### 2.5 Histology

Hematoxylin and eosin (H&E) staining was performed according to previous study (25). Liver and epididymal adipose tissues were fixed in 10% neutral formalin for 24 h and processed for H&E staining after embedding the tissues in conventional paraffin. Variation in liver and epididymal adipose tissue morphology and pathology between groups was then observed under a light microscope at 40× magnification (Nikon, Japan).

### 2.6 Gut microbiota composition analysis

Changes in the composition of the gut microbiota were determined by using 16S rRNA gene sequencing analysis. Mouse fecal samples were collected, and DNA was extracted using an E.Z.N.A. Bacterial DNA Extraction kit (Omega Bio-tek, Norcross, GA, United States). Amplification of the V4–V5 region of the bacterial 16S rRNA genes was performed using the universal primers 515F (5′-barcode-GTGCCAGCMGCCGCGG-3′) and 907R (5’-CCGTCAATTCMTTTRAGTTT-3′). After amplification, samples were sent to Shanghai Majorbio Biomedical Technology, Co., Ltd., for sequencing. The impacts of RSG on gut microbiota composition were then investigated based on α-diversity, β-diversity, genera taxonomic composition, and Phylogenetic Investigation of Communities by Reconstruction of Unobserved States (PICRUSt2) analysis.

### 2.7 Fecal metabolome

Fecal metabolic profiles were assessed using a UHPLC-Q Exactive HF-X system (Thermo Fisher Scientific) equipped with an ACQUITY HSS T3 column (2.1 × 100 mm, 1.8 μm; Waters, Milford, MA, United States) under analytical conditions similar to previously described methods (26). Data were acquired under data-dependent acquisition mode and were analyzed in SIMCA 15.0 by using principal component and partial least-squares discriminant analyses (PCA and PLS-DA, respectively). An orthogonal partial least squares-discriminant analysis (OPLS-DA) was used for screening metabolites that demonstrated significant differences in abundance between the MOD and RSG-H groups (VIP value >1.0, *p* < 0.05). Metabolite identification was performed using the Human Metabolome Database,^1^ and MetaboAnalyst 5.0 was used to analyze metabolic pathways of interest that demonstrated significant differences between groups.

### 2.8 Data analysis and visualization

The animal experimental data of body weight, serum lipid, ALT, and AST levels were obtained from 6 mice per group (*n* = 6) and were presented as means ± standard deviation (SD). To determine statistical significance, data were analyzed by one-way analysis of variance (ANOVA) followed by a least significant difference (LSD) *post hoc* test; the IBM SPSS Statistics 21 platform were used to perform statistical analysis and a *p-*value cutoff of <0.05 was used. All different superscript letters in figures indicate statistically significant differences between groups.

## 3 Results

### 3.1 Glucosinolate composition in RSG extract

**Figure 1:**
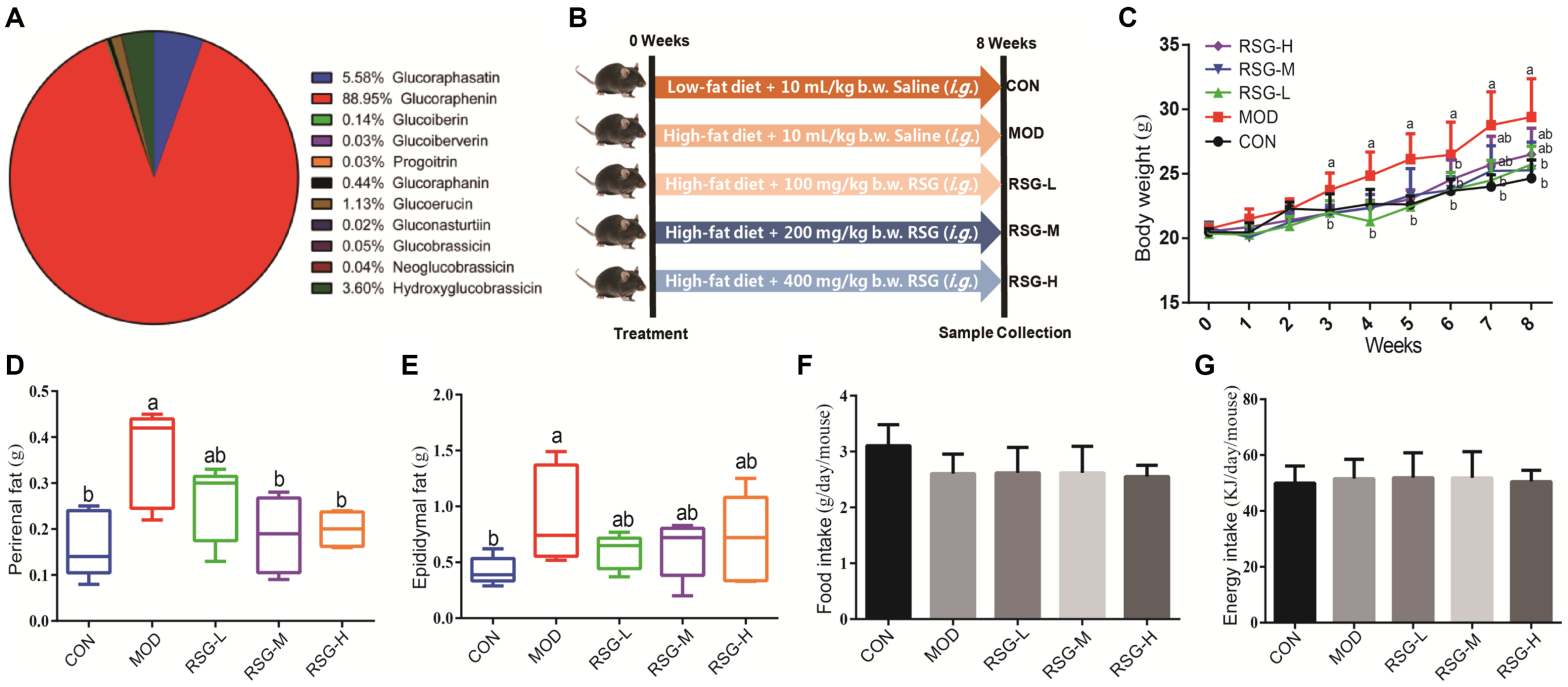
Effect of RSG extract on weight and food consumption in obese mice fed an HFD. **(A)** Glucosinolate composition in RSG extract. **(B)** Experimental design. **(C)** Whole body weight. **(D)** Weight of perirenal fat tissue. **(E)** Weight of epididymal fat tissue. **(F)** Food consumption. **(G)** Energy intake. The different superscript letters indicate significant differences (*p* < 0.05) among different groups. Statistical analysis was performed using one-way ANOVA (*post hoc* test).

In total, 11 glucosinolate substances in RSG extract were detected, of which the most dominant glucosinolate was glucoraphenin (accounting for 88.95% of the total glucosinolate content), followed by glucoraphasatin (5.58%) and hydroxyglucobrassicin (3.60%; Figure 1A). This finding is consistent with previous research that showed the glucoraphenin (84.4–92.8%) was the most abundant glucosinolate in radish seed (27).

### 3.2 RSG extract reduces body weight and fat mass in obese mice

We established a mouse model of obesity to study the impacts of RSG extract on body weight and fat mass (Figure 1B). MOD group that were fed an HFD demonstrated a 17.12% increase in body weight compared with the CON group. However, obese mice treated for 8 weeks with RSG extract demonstrated significant reductions in body weight compared with MOD obese mice, regardless of dose of RSG administered (Figure 1C). In agreement with this finding, MOD obese mice demonstrated greater perirenal and epididymal fat mass than the CON group, and all doses of RSG significantly inhibited the accumulation of this adipose tissue (Figures 1D,E). In addition, MOD obese mice fed an HFD appeared to eat less than CON group; however, this comparison did not reach statistical significance, and no significant differences in average daily food consumption were found between all groups (Figures 1F,G). Together, these results suggest that RSG extract reduced body weight gain and adipose accumulation by HFD, potentially attenuating obesity.

### 3.3 RSG extract lowers serum lipid and liver enzyme levels in obese mice

**Figure 2:**
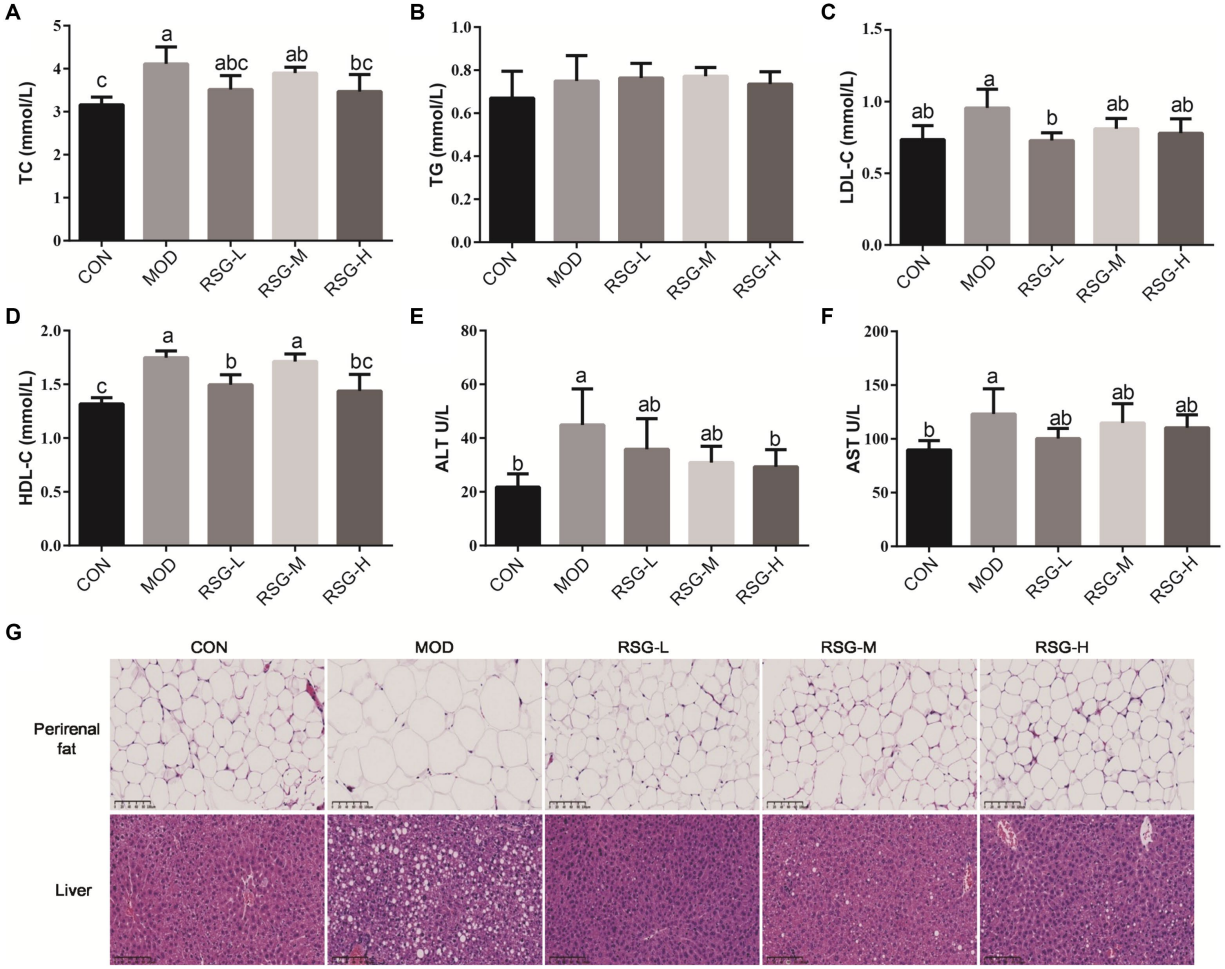
Effect of RSG extract on serum lipid, ALT, and AST levels and histopathology of liver and epididymal adipocytes in obese mice fed an HFD. **(A–F)** Mouse sera were assessed for differences in concentrations of TC **(A)**, TG **(B)**, LDL-C **(C)**, HDL-C **(D)**, ALT **(E)**, and AST **(F)**. **(G)** Histopathology of liver and epididymal adipocytes (40× magnification). The different superscript letters indicate significant differences (*p* < 0.05) among different groups. Statistical analysis was performed using one-way ANOVA (*post hoc* test).

The MOD obese mice demonstrated higher levels of serum TG, TC, LDL-C, and HDL-C than CON group, and differences in TC and LDL-C levels were statistically significant (Figures 2A–D). All doses of RSG extract reduced serum lipid levels compared with MOD obese mice, in particular TC and LDL-C. Notably, LDL-C levels in obese mice treated with RSG reached the same level as that observed in the CON group (Figure 2C).

Mice fed an HFD demonstrated increased levels of serum ALT and AST compared with the CON group (2.13-fold and 1.38-fold higher, respectively; Figures 2E,F), which is similar to findings observed in previous studies that mice fed an HFD long term develop liver damage (28). Interestingly, administration of RSG extract at all doses significantly reduced serum ALT and AST levels in obese mice, and ALT levels observed in the RSG-H group mirrored those observed in the CON group, suggesting that RSG extract has a protective effect on the liver.

### 3.4 RSG extract decreases lipid deposition in obese mice

Lipid deposition in liver and epididymal adipocytes was evaluated by H&E staining. Epididymal fat cells in CON group mice demonstrated a small and tightly arranged morphology, whereas epididymal fat cells in mice fed an HFD were significantly larger (Figure 2G). However, fat cell size was significantly decreased after treatment with RSG extract, regardless of dose, and fat cell morphology was close to that observed in the CON group. H&E staining of liver tissues demonstrated that MOD obese mice had higher numbers of lipid droplets than the CON group, and MOD obese mice exhibited hepatocellular steatosis and vacuolization of hepatocytes. After 8 weeks of treatment with RSG extract, lipid accumulation in the livers of obese mice was significantly improved, and hepatocytes lacked the presence of lipid droplets.

### 3.5 Composition of the gut microbiota after RSG extract intervention

**Figure 3:**
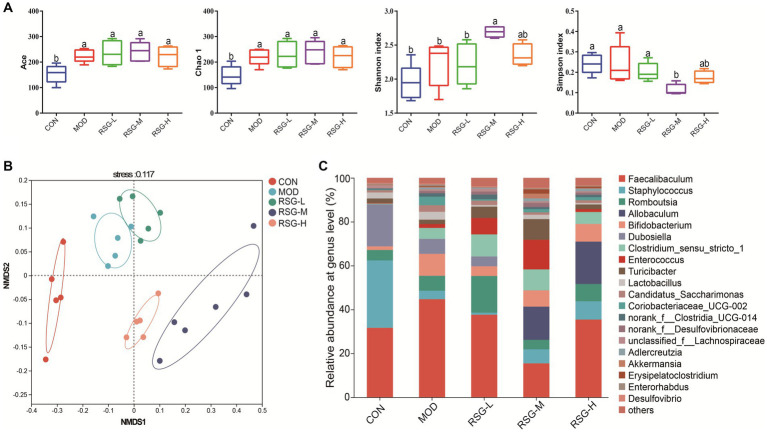
Composition of the gut microbiota in obese mice fed an HFD after treatment with RSG extract. **(A)** α-Diversity (Ace, Chao 1, Shannon, and Simpson indexes). **(B)** NMDS based on the Bray–Curtis distance algorithm. **(C)** Gut microbiota composition at the genus level. The different superscript letters indicate significant differences (*p* < 0.05) among different groups. Statistical analysis was performed using one-way ANOVA (*post hoc* test).

To investigate the role of RSG extract in regulating the gut microbiota, we used 16S rRNA gene sequencing to determine the different bacteria present in the gut microbiome. First, α-Diversity, as determined using the Simpson, Shannon, Chao1, and Ace indexes, was used to assess gut microbiota abundance and diversity. Chao1 and Ace indexes of mice in each group treated with RSG tended to increase compared with the Chao1 and Ace indexes of MOD obese mice, but no statistically significant difference was observed. Additionally, the Shannon indexes of obese mice treated with medium and high concentrations of RSG were significantly higher than that observed in MOD obese mice, and the Simpson index was significantly lower (Figure 3A), suggesting that treatment with RSG extract increases gut microbiota diversity in obese mice. We next used non-metric multidimensional scaling (NMDS) based on Bray–Curtis distance to assess β-diversity. Using this method, we observed that the gut microbiota of MOD obese mice and CON group were distinctly different from each other, suggesting that an HFD could trigger gut microbiota dysbiosis (Figure 3B). After treatment with RSG extract, regardless of dose, the composition of the gut microbiota was distinctly different from the gut microbiota observed in MOD obese mice, but this effect was dose dependent. Specifically, the gut microbiota from obese mice treated with medium and high doses of RSG were completely separated from the gut microbiota from MOD obese mice.

**Figure 4:**
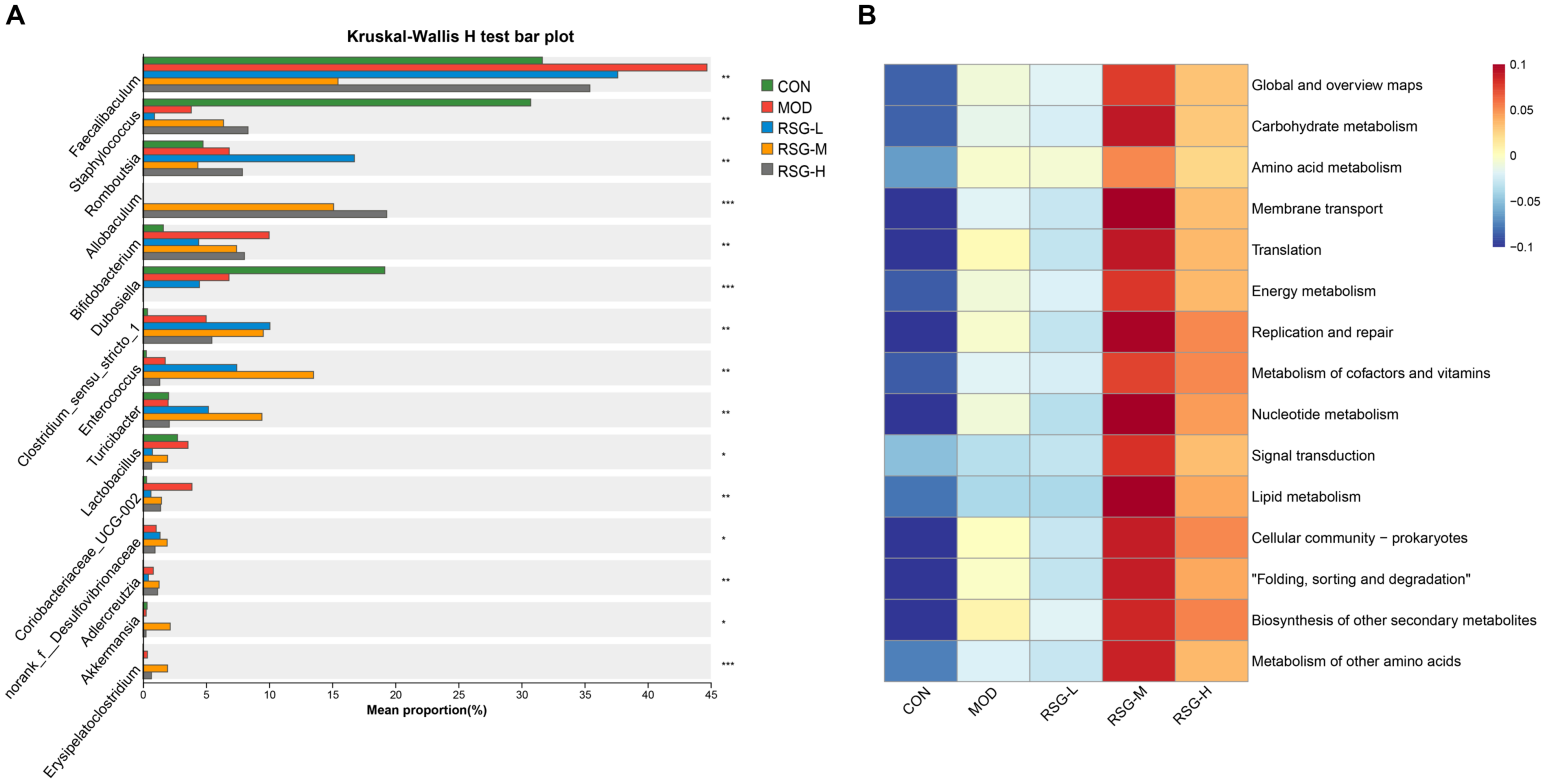
**(A)** Comparison of significantly changed genera among the CON, MOD, RSG-L, RSG-M, and RSG-H groups. **(B)** Functional prediction (PICRUSt) of the gut microbiota based on KEGG pathways in obese mice and in obese mice treated with RSG extract.

When examining the composition of the gut microbiota at the genus level, *Faecalibaculum*, *Staphylococcus*, *Romboutsia*, *Allobaculum*, *Bifidobacterium*, *Dubosiella*, *Clostridium sensu stricto*, and *Enterococcus* were the most common genera in each group, among which *Faecalibaculum* was the main dominant bacteria genus (Figure 3C). The abundance of *Faecalibaculum*, *Clostridium sensu strict*, and *Bifidobacterium* in MOD obese mice was increased compared with that observed in the CON group, whereas the abundance of *Staphylococcus* and *Dubosiella* was decreased (Figure 3C). In comparison with MOD obese mice, the abundance of *Allobaculum*, *Romboutsia*, *Enterococcus*, *Turicibacter*, and *Akkermansia* was increased and the abundance of *Faecalibaculum* and *Dubosiella* was decreased after RSG treatment (Figure 4A). Among the three different doses of RSG groups, RSG-L had the highest abundances of *Romboutsia*. Notably, *Enterococcus*, *Turicibacter*, and *Akkermansia* were the most abundant in RSG-M group. Meanwhile, RSG-H had the highest abundances of *Allobaculum*.

Furthermore, to determine how shifts in the gut microbiota may functionally impact metabolism, a PICRUSt2 analysis was performed based on the KEGG database. The top 15 abundant functions were selected, and abundance information in the different groups was plotted as a heat map (Figure 4B). Global and overview maps, carbohydrate metabolism, amino acid metabolism, membrane transport and energy metabolism were the major metabolic functional activities of the gut microbiota in this study. The functions of carbohydrate metabolism, amino acid metabolism, energy metabolism and lipid metabolism were enriched in obese mice treated with medium and high doses of RSG compared with in MOD obese mice. These findings suggest that treatment with RSG extract improves energy and lipid metabolism, which may aid in preventing obesity.

### 3.6 Impacts of RSG extract on the fecal metabolome of obese mice

**Figure 5:**
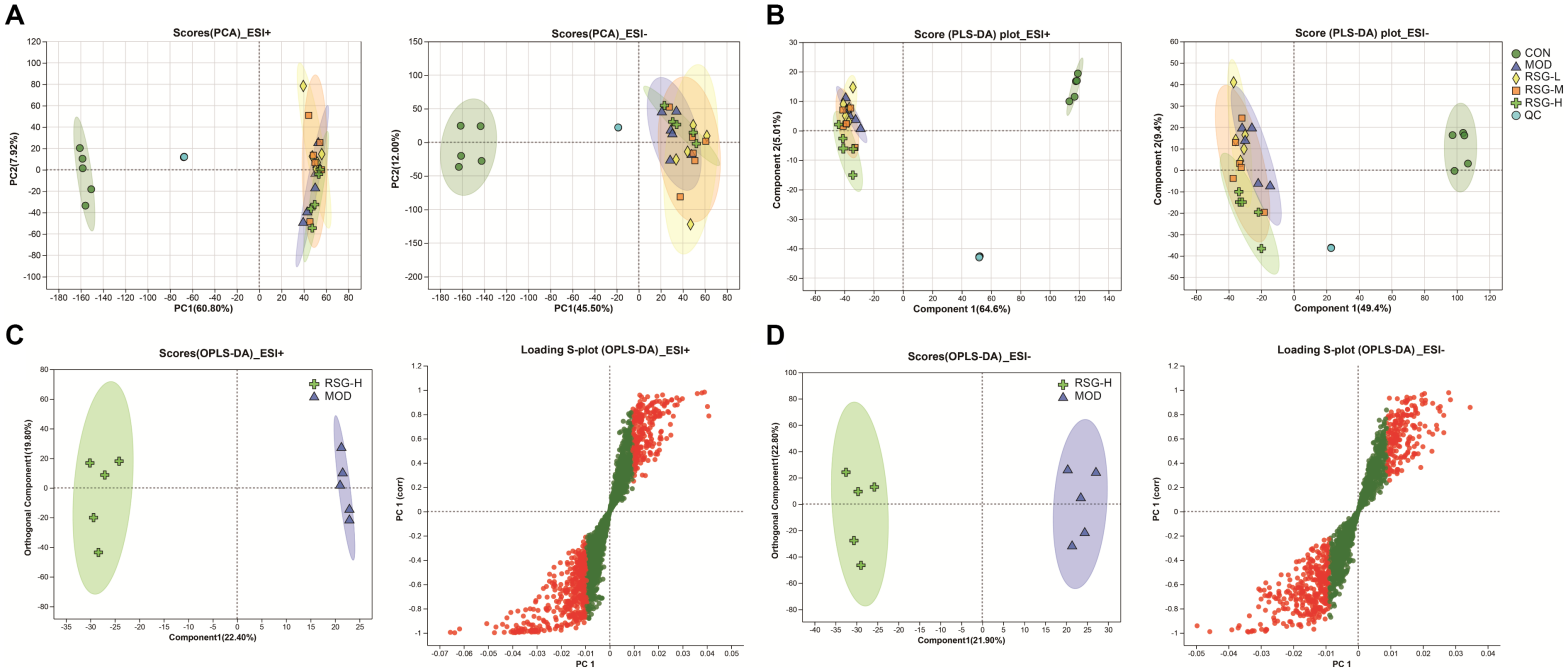
The fecal metabolome of obese mice treated with RSG extract. **(A)** PCA score plots of the detected metabolites among CON, MOD, RSG-L, RSG-M, and RSG-H groups. **(B)** PLS-DA score plots of the detected metabolites among CON, MOD, RSG-L, RSG-M, and RSG-H groups. **(C,D)** OPLS-DA between the MOD and RSG-H groups. Data from positive ion mode **(C)** and negative ion mode **(D)** are depicted.

In the present study, fecal metabolic profiles were assessed using untargeted metabolomics to gain further insight into the anti-obesity effects of RSG extract. In total, 2,532 metabolites were detected under positive ion mode, and 1,810 metabolites were identified under negative ion mode in all samples. When analyzed by PCA, the metabolic profiles of the CON and MOD groups were completely unique, whereas the metabolic profiles of the RSG-L, RSG-M, and RSG-H groups and the MOD group showed partial overlap (Figure 5A). Further PLS-DA results showed that obese mice treated with RSG gradually deviated from the metabolic profiles observed in MOD obese mice, especially the RSG-H group, which was completely separated from the MOD group, indicating that high doses of RSG extract could change the fecal metabolome of obese mice (Figure 5B). Therefore, the RSG-H group was chosen to screen for differential metabolites compared with the MOD group. We observed a complete separation between MOD obese mice and mice treated with the high dose of RSG, as determined using an OPLS-DA, with *R*^2^ values of 0.9024 (positive ion mode) and 0.9753 (negative ion mode) and *Q*^2^ values of −0.0854 (positive ion mode) and −0.0084 (negative ion mode), indicating model stability and reliability (Figures 5C,D).

**Figure 6:**
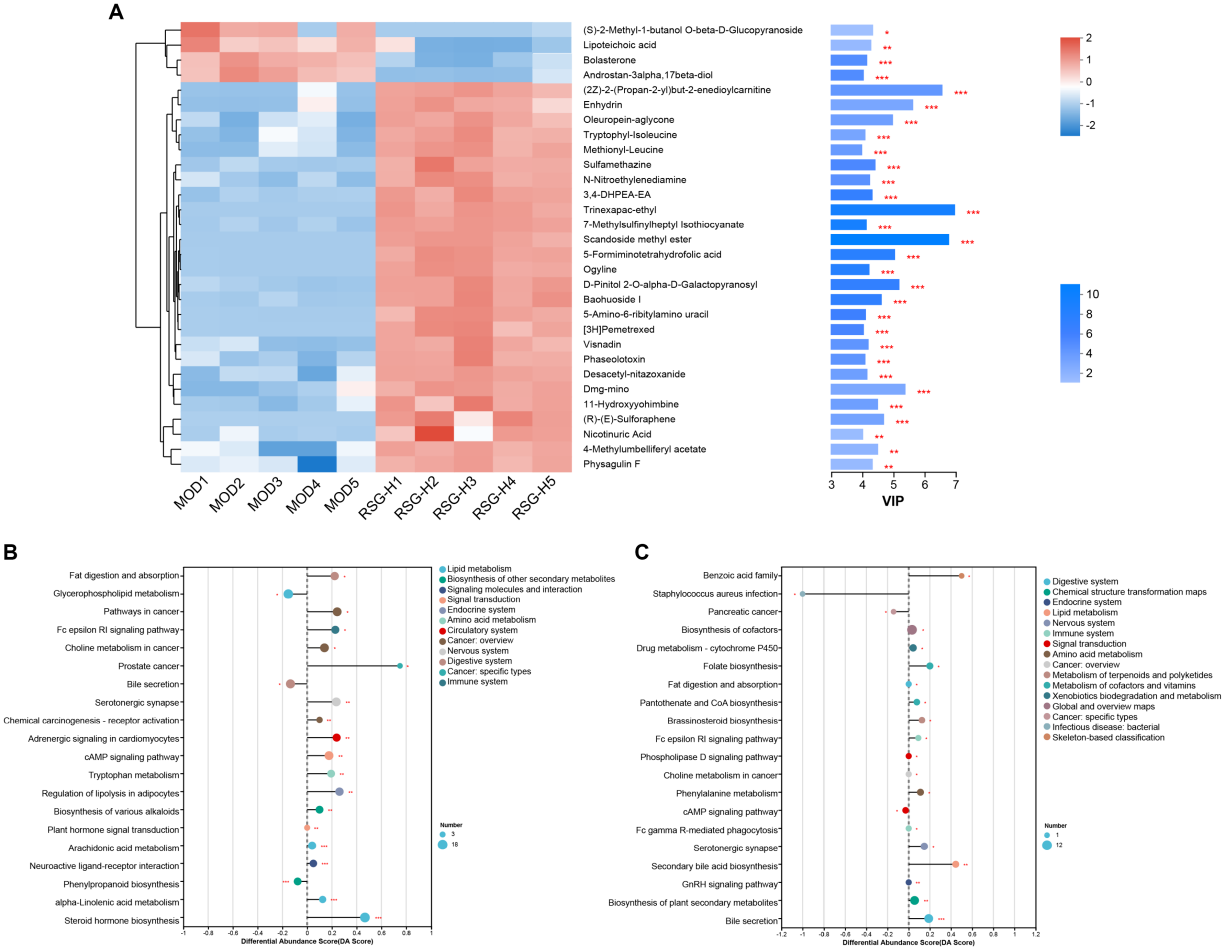
RSG extract treatment changes the fecal metabolome of obese mice fed an HFD. **(A)** Expression of the top 30 differentially abundant metabolites. **(B,C)** Metabolic pathway analysis between CON and MOD groups **(B)** and between MOD and RSG-H groups **(C)**.

Differential fecal metabolite abundance observed between the MOD and RSG-H groups was screened out using the standards of a VIP of ≥1 and a *p-*value of <0.05. In total, 570 metabolites were screened, of which 363 were increased in abundance and 207 were decreased in abundance in the RSG-H group. We next analyzed variation in the top 30 differentially expressed metabolites between the MOD and RSG-H groups (Figure 6A). Mice in the RSG-H group demonstrated a significant upregulation in 26 specific metabolites compared with the MOD group, such as trinexapac-ethyl, scandoside methyl ester, enhydrin, sulforaphene, and 7-methylsulfinylheptyl isothiocyanate, whereas the four metabolites of (*S*)-2-methyl-1-butanol *O*-*β*-d-glucopyranoside, lipoteichoic acid, bolasterone, and androstan-3α-17β-diol were downregulated (Figure 6A). To further understand the metabolic alterations in obese mice after treatment with RSG extract, metabolic pathway enrichment analysis was performed (Figures 6B,C). Treatment with RSG extract significantly changed the pathways of bile secretion, biosynthesis of plant secondary metabolites, secondary bile acid biosynthesis, serotonergic synapse, and fat digestion and absorption (Figure 6C).

**Figure 7:**
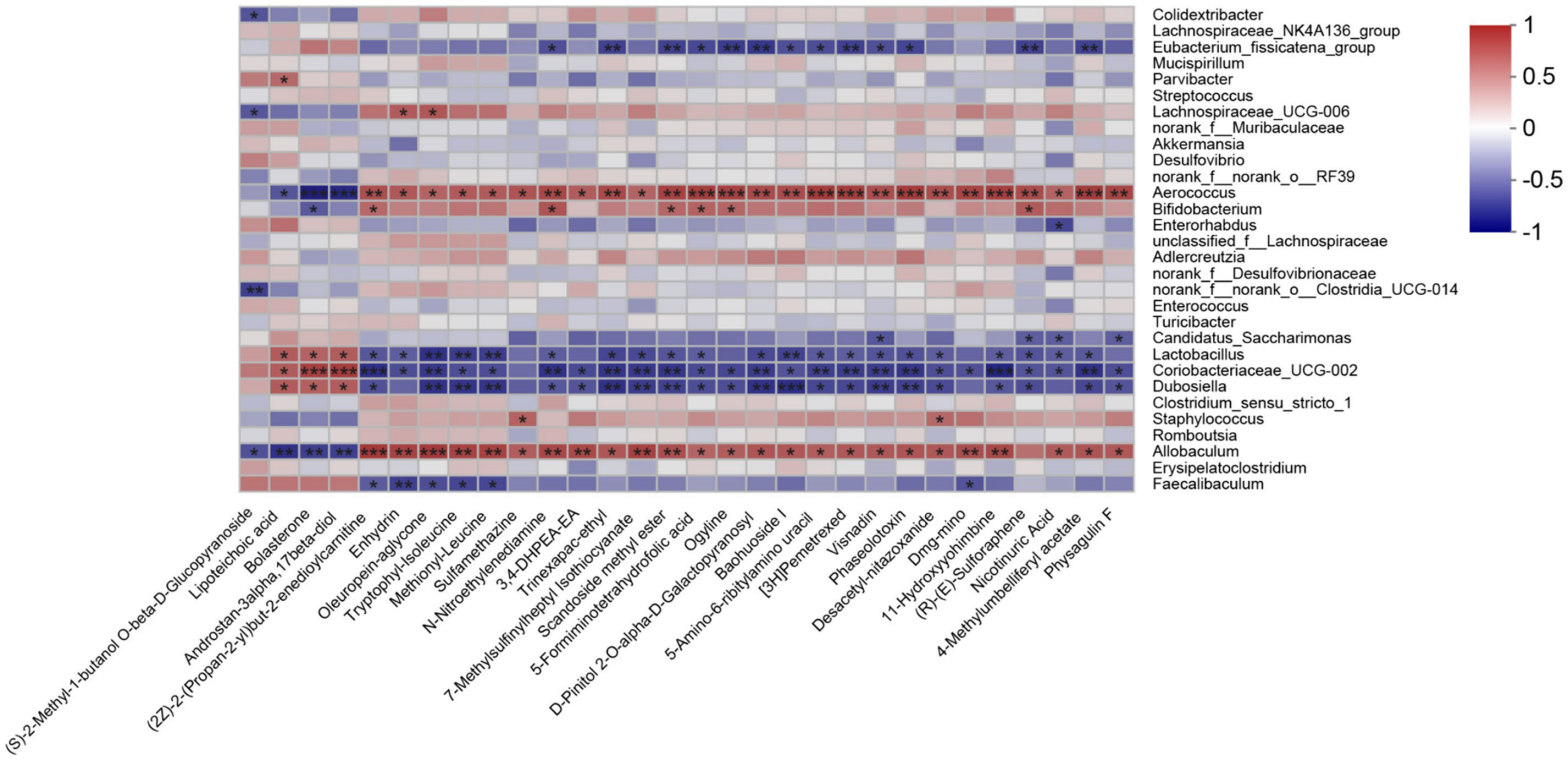
Heatmap of the Spearman’s correlation analyses between top 30 differentially expressed fecal metabolites and gut microbial genera (top 30). ^*^p < 0.05, ^**^p < 0.01, ^***^p < 0.001.

To study the association between the gut microbiota and different metabolites, a Spearman correlation analysis on the differentially expressed fecal metabolites and the genus-level gut microbiota data was performed. *Allobaculum* was positively associated with most of the upregulated metabolites in the RSG-H group (Figure 7). Moreover, the presence of *Bifidobacterium* was positively correlated with sulforaphene, 5-formiminotetrahydrofolic acid, scandoside methyl ester, and *N*-nitroethylenediamine. In addition, *Lachnospiraceae_UCG-006* was positively correlated with the presence of enhydrin and oleuropein-aglycone; however, the abundance of *Lactobacillus*, *Coriobacteriaceae_UCG-002*, and *Dubosiella* was negatively correlated with metabolites that were upregulated in the RSG-H group (Figure 7).

## 4 Discussion

Obesity is typically caused by excessive energy intake or abnormal accumulation of fat as a result of an imbalance between energy intake and energy expenditure accompanied by different degrees of metabolic disorders (29). Obesity is a global public health problem that not only leads to the development of chronic diseases such as type 2 diabetes, hypertension, and fatty liver but also increases the economic burden on society and patients (30). With advancements in biomedical research, studies have found that natural medicines and functional foods have the advantage of targeting multiple components and pathways associated with the development of chronic metabolic diseases, and have fewer toxicities and side effects (4, 31). Therefore, plant-derived functional foods that rich in bioactives have been recognized as a promising and viable alternative method for preventing and alleviating obesity.

Radish seed is a functional food with the potential to prevent metabolic diseases (12). Previous research has shown that aqueous extract of radish seeds can regulate lipid metabolism by inhibiting fat formation and accelerating fat decomposition, and the obesity-alleviating effects of radish seeds are probably due to the presence of appreciable amounts of glucosinolates (10). Therefore, we established an obese mouse model by feeding C57BL/6 mice an HFD and monitoring body weight, serum lipid levels, the gut microbiota, and the fecal metabolome after treatment with RSGs, to investigate the obesity-alleviating effects of RSG extract. The results showed that RSG extract had a clear inhibitory effect on the development of obesity in mice and significantly reduced body weight and serum lipid levels (TG, TC, and LDL-C) and liver enzymes (ALT and AST) in obese mice. In addition, H&E staining of the liver showed decreased numbers of lipid droplets, and adipocyte volume and number were also significantly reduced in adipose tissue, suggesting that RSG extract can alleviate lipid accumulation. Moreover, food consumption was monitored regularly, and administration of RSG extract did not affect appetite, which further confirmed that the weight loss and lipid-lowering effects of RSG extract have no connection with food intake.

Imbalances in the gut microbiota are related to the development of chronic metabolic diseases, such as obesity, diabetes, and cardiovascular disease (17, 32). We found that the weight loss and lipid-lowering effects of RSG extract were accompanied by significant changes in the gut microbiota. A clear separation between the gut microbiota from the MOD group and the gut microbiota from the groups treated with RSG extract (especially the RSG-H group) was observed (Figure 3B). At genus level, we found that the abundances of the genera *Faecalibaculum* and *Clostridium sensu stricto* in MOD obese mice were significantly higher than those observed in the CON group, whereas the relative abundances of the genera *Staphylococcus* and *Dubosiella* were lower. After treatment with RSG extract, the abundances of the genera *Faecalibaculum* and *Dubosiella* were downregulated, and the abundances of *Allobaculum*, *Romboutsia*, *Enterococcus*, *Turicibacter*, and *Akkermansia* were upregulated (Figure 4A). Previous research has shown that *Faecalibaculum* is significantly enriched in obese mice (33), supporting our findings in mice in the MOD group. The shift from a higher abundance of *Faecalibaculum* to a higher abundance of *Allobaculum*, *Romboutsia*, *Turicibacter*, and *Akkermansia* after treatment with RSG extract is of interest because the above-mentioned bacteria are beneficial to anti-obesity. *Allobaculum* and *Akkermansia*, which were identified as beneficial genus to host health, have been widely investigated for obesity mitigation (34). Besides, *Allobaculum* is also involved in the biotransformation of phytochemicals in the intestine; for example, the presence of *Allobaculum* is correlated with the biotransformation of nobiletin and enhances its antiobesity activity (35). Consistently with these previous studies, a remarkable higher abundance of *Allobaculum* and *Akkermansia* was found in obese mice supplemented with RSG in this study, especially in RSG-M and RSG-H groups. Moreover, *Romboutsia* was also shown to be negatively correlated with obesity-related indexes (35), and the RSG-L group demonstrated a 9.91% increase in the relative abundance of *Romboutsia* when compared with the MOD obese mice in this study. In a high-fat diet-induced obese mice model, Quan et al. (36) fund that *Turicibacter* activated the thermogenic activity of brown adipose tissue and activates the formation of beige adipose tissue in obese mice fed an HFD, ultimately reducing fat accumulation and alleviating obesity. Additionally, *Turicibacter* was also classified as intestinal probiotic by production of SCFAs, thereby alleviating obesity (37). In line with the mentioned previous studies, we also observed that RSG supplementation led to an increase in the abundance of *Turicibacter*. These results suggested that the anti-obesity effect RSG might be correlated with altered gut microbiota, which could facilitate the growth of beneficial genera and suppress the obesity-related bacteria. Furthermore, the PICRUSt2 function prediction results showed that the abundance of specific bacteria in the gut microbiota that contribute to carbohydrate metabolism, amino acid metabolism, energy metabolism, and lipid metabolism was increased after treatment with RSG extract in obese mice, indicating that RSG could help cultivate a gut microbiota that facilitates metabolism and alleviates obesity.

Glucosinolates are a group of characteristic secondary metabolites which present in all parts of Cruciferous vegetable, such as broccoli, cauliflower, cabbage, and radish (38–41). Several previous studies have demonstrated that the presence of appreciable amounts of glucosinolates (mainly glucoraphanin and glucoraphenin) in radish-based diet contribute to its health benefits (10, 42). In the current study, we found glucoraphenin and glucoraphasatin are the dominant glucosinolate in radish seed, which accounting for 88.95 and 5.58% of the total glucosinolate content. However, glucosinolate itself is not bioactive and requires degradation by vegetal myrosinase or microbial metabolism to generate isothiocyanates to be bioactive (43, 44). The isothiocyanates produced by glucosinolate degradation have numerous physiological functions, such as improving glycolipid metabolism, inhibiting inflammation, and preventing cancer (38–40).

Plant myrosinase is highly sensitivity to thermal processing, and more than 90% of myrosinase activity was lost after 5 min of treatment at 60°C (45). Usually, radish seeds should be used after stir-frying under high temperature; as a result, glucosinolates in dried radish seeds cannot be hydrolyzed by plant enzymes due to the loss of myrosinase activity during stir-frying (10). Therefore, the degradation of RSG *in vivo* is likely catalyzed by intestinal flora. We demonstrated that the fecal metabolome of obese mice were significantly altered after RSG treatment, and the difference between the fecal metabolome in the RSG-H and MOD groups was very significant. Fecal metabolites such as sulforaphene and 7-methylsulfinylheptyl isothiocyanate were significantly upregulated in the RSG-H group (Figure 6A). Notably, sulforaphene is the isothiocyanate generated by degradation of glucoraphenin (the most dominant glucosinolate in radish seeds). Sulforaphene has antiobesity effects, can inhibit adipocyte differentiation via Hedgehog signaling, and inhibits fat accumulation in 3T3-L1 precursor adipocytes by promoting oxidative lipolysis (46, 47). Research on the transformation of glucosinolate *in vitro* showed that *Allobaculum*, *Enterococcus* and *Bifidobacterium*, which are increased in abundance in the gut microbiota after treatment with RSG extract, can degrade glucosinolates to produce isothiocyanate (48, 49). Correlation analysis between gut microbiota and fecal metabolites showed that *Bifidobacterium* was positively correlated with sulforaphane abundance, and *Allobaculum* was positively correlated with the levels of 7-methylsulfinylheptyl isothiocyanate. As a result, we speculated that RSG could be degraded by gut microbiota to generate isothiocyanate-like products, thereby alleviating obesity. Overall, we show that the obesity-alleviating effects of RSG are mediated, at least in part, by altering the gut microbiota and increasing the abundance of beneficial genera (including *Allobaculum* and *Bifidobacterium*), which generate isothiocyanates such as sulforaphene and upregulate metabolism-related pathways such as energy metabolism and lipid metabolism, in turn facilitating weight loss and lowering circulating lipid levels.

## 5 Conclusion

Glucosinolates are the main bioactive components in radish seed. Treatment with radish seed glucosinolates (RSGs) for 8 weeks could effectively prevent weight gain; lower serum lipid, AST, and ALT levels; and lower lipid accumulation in liver and epididymal adipocytes in obese mice fed an HFD. The weight loss and lipid-lowering effects of RSG are related to differential regulation of the gut microbiota, which thereby impacts the degradation rates of glucosinolates, the generation of isothiocyanates such as sulforaphene and 7-methylsulfinylheptyl isothiocyanate, and the upregulation of related metabolic pathways such as energy metabolism and lipid metabolism. Here, we show the utility of natural active components in the prevention and treatment of chronic metabolic diseases such as obesity to expand the application and promotion of radish seeds.

## Data availability statement

The original contributions presented in the study are included in the article/supplementary material, further inquiries can be directed to the corresponding authors.

## Ethics statement

The animal study was approved by the Animal Ethics Committee of Zhejiang University of Technology (ZH20230904008). The study was conducted in accordance with the local legislation and institutional requirements.

## Author contributions

QZ: Data curation, Methodology, Software, Visualization, Writing – original draft. PZ: Data curation, Methodology, Writing – original draft. DL: Data curation, Investigation, Methodology, Writing – review & editing. LT: Conceptualization, Methodology, Validation, Writing – review & editing. JY: Conceptualization, Software, Writing – original draft. CZ: Conceptualization, Funding acquisition, Writing – review & editing. GJ: Conceptualization, Funding acquisition, Supervision, Writing – review & editing.

## Footnotes

^1^ http://www.hmdb.ca/
